# Insulin negatively regulates dedifferentiation of mouse adipocytes in vitro

**DOI:** 10.1080/21623945.2020.1721235

**Published:** 2020-01-28

**Authors:** Liguo Zang, Suchart Kothan, Yiyi Yang, Xiangyi Zeng, Lingmin Ye, Jie Pan

**Affiliations:** a Shandong Provincial Key Laboratory of Animal Resistant Biology, College of Life Sciences, Shandong Normal University, Jinan, China; b Center of Radiation Research and Medical Imaging, Department of Radiologic Technology, Faculty of Associated Medical Sciences, Chiang Mai University, Chiang Mai, Thailand

**Keywords:** Insulin, adipocyte dedifferentiation, OSI-906, PI-3K, AKT

## Abstract

Insulin plays an important role during adipogenic differentiation of animal preadipocytes and the maintenance of mature phenotypes. However, its role and mechanism in dedifferentiation of adipocyte remains unclear. This study investigated the effects of insulin on dedifferentiation of mice adipocytes, and the potential mechanisms. The preadipocytes were isolated from the subcutaneous white adipose tissue of wild type (WT), TNFα gene mutant (TNFα-/-), leptin gene spontaneous point mutant (*db/db*) and TNFα-/-/*db/db* mice and were then induced for differentiation. Interestingly, dedifferentiation of these adipocytes occurred once removing exogenous insulin from the adipogenic medium. As characteristics of dedifferentiation of the adipocytes, downregulation of adipogenic markers, upregulation of stemness markers and loss of intracellular lipids were observed from the four genotypes. Notably, dedifferentiation was occurring earlier if the insulin signal was blocked. These dedifferentiated cells regained the potentials of the stem cell-like characteristics. There is no significant difference in the characteristics of the dedifferentiation between the adipocytes. Overall, the study provided evidence that insulin plays a negative regulatory role in the dedifferentiation of adipocytes. We also confirmed that both dedifferentiation of mouse adipocytes, and effect of the insulin on this process were independent of the cell genotypes, while it is a widespread phenomenon in the adipocytes.

## Introduction

Dedifferentiation of cells is a reversal of the developmental process, such as in cases of differentiated mature cells with specialized functions that produce undifferentiated progenitor cells [1–3] or they undergo a self-repair mechanism [4,5] under certain physiological and/or pathological conditions. Spalding et al. reported that approximately 10% of adipocytes in human white adipose tissue are renewed annually during adult age [1]. However, it remains unclear whether these new mature adipocytes originated from stem cells, or were derived partially from mature adipocytes that undergo a renewal cycle, i.e. mature adipocyte → dedifferentiation→proliferation→ adipogenic redifferentiation, thereby producing more adipocytes. It is reported that reducing body weight by 5–10% can improve blood lipid abnormality and insulin resistance in humans [6]. Since hyperproliferation and hypertrophy of adipocytes are a typical phenomenon in overweight individuals and obese patients, induction and promotion of dedifferentiation of adipocytes within a controlled range may become a strategy to prevent and reduce such abnormal phenomena of adipocytes. On the other hand, dedifferentiated cells are considered to be an attractive cell source that can be used in regenerative medicine and cell therapy field [3,7,8].

Recent studies have shown that a dedifferentiation was observed in primary mature adipocytes when they were cultured in common cell growth medium, and the same phenomenon also happened in differentiated adipocytes in vitro [3,9,10]. The mature adipocyte markers were down-regulated slowly, while cell cycle and extracellular matrix remodelling molecules were up-regulated slowly, followed by gradual loss of intracellular lipids. These cells can restore the ability to resemble pluripotent stem cells, such as the ability to redifferentiate to adipocytes and/or transdifferentiate into other cell types [7,8,11,12]. This reflects the plasticity change characteristics of the mature adipocytes phenotype, i.e. adipocytes can transform their phenotype between maturation and stem cell-like undifferentiation. Remarkably, the dedifferentiation of adipocytes only occurs when they were cultured in common cell growth medium (without adipogenic inducer), which indicates that some regulatory factors exist in the cell culture microenvironment that influences the process of cell dedifferentiation. Based on the data from humans [9], animals [7,8,12] and our previous experiments, the following conjectures can be made: 1) the commonly used cell culture environment is a trigger factor of adipocyte phenotype switching, and 2) certain key factors in the maintenance of the mature phenotype of adipocytes exist in the in vivo environment (such as serum) and adipogenic differentiation medium, but they were absent in the common cell growth medium. Insulin may be a key regulatory factor in the process of dedifferentiation of adipocyte since it is a major and indispensable inducer for adipogenesis both in vivo and in vitro. Insulin, as a growth factor and lipogenic and antilipolytic peptide hormone, plays an important role in cell proliferation, adipogenic differentiation and glucose-lipid metabolism [13–15]. To our knowledge, the question as to how insulin/insulin signal controls mature adipocyte dedifferentiation, and whether the effect of insulin on adipocyte dedifferentiation is correlated with cell genotypes remains unclear. To answer these questions, the insulin signal inhibition experiment was designed in this study, and the characteristics of the dedifferentiation of four genotypes of mice adipocytes were comparatively investigated. Our data indicated that the insulin signal plays a negative control for dedifferentiation of adipocytes, also its effect on the process is independent of the cell genotypes.

## Materials and methods

### Primary mouse preadipocytes isolation and culture

C57BL/6 TNFα knockout (TNFα-/-) mice and leptin receptor spontaneous heterogeneous mutate (Lepr*^db/+^, db/+*) mice were purchased from the Jackson Laboratory (Bar Harbour, USA). Homogeneous Lepr*^db/db^* (*db/db*) mice were obtained by the inbreeding of *db/+* mice. The *db/+* and TNFα-/- mice were further crossbred to obtain *db/db* and TNFα double mutant (DT) mice. The genotypes of these mice were confirmed by PCR. C57BL/6 wild-type (WT) mice were purchased from Shanghai SLAC Laboratory Animal Co., Ltd. (Shanghai, China) as the normal control. Six- to eight-week-old male mice of each genotype (n ≥ 7, each time) were used to isolate primary preadipocytes. Briefly, mice epididymal white adipose tissues were removed and digested in 0.1% (v/v) type I collagenase solution (containing 0.4% BSA, v/v) in a 37°C water bath with shanking at 100 rpm for 35 min. After adding cell growth medium (DMEM/F12 containing 10% FBS (v/v), 100 U/ml penicillin and 100 µg/ml streptomycin) was used to stop the digestion. The suspension was centrifuged at 200 × g for 10 min, and then the cell pellet was resuspended in a cell growth medium, was filtered through a 100-µm strainer, and was seeded in 25-cm2 flasks. Mesenchymal stromal cells do not easily to attach to the bottom of a petri dish after seeding when compared to fibroblasts, but they are more sensitive to trypsin when passage digestion takes place, and they are easy to de-attach from dish. Therefore, we use differential adherence and incomplete digestion methods to improve the purity of mesenchymal stromal cells until they were at passage 4 (P4) to be subcultured into 12-well plates. These preadipocytes were then used for further experiments.

### Adipogenic differentiation

The preadipocytes from four genotypes of mice were used to induce adipogenic differentiation in vitro, respectively. Briefly, 2 × 10^4^ cells/cm^2^ of the cells were subcultured in 12-well plates in a cell growth medium. Two days after the cells were confluent (adipogenic differentiation day 0, D0) they were induced for adipogenesis using inducing cocktail medium (MDI, cell growth medium supplemented with 17 nM insulin, 1 µM Dex and 0.25 mM IBMX; all from Sigma-Aldrich). Three days later (D3), the adipogenic inducing MDI medium was replaced with adipogenic maintenance medium (cell growth medium supplemented with 17 nM insulin) and was further induced continually for 5 d in order to completely induce adipogenic differentiation.

### Dedifferentiation of the adipocytes

Adipogenic maintenance medium was replaced by a cell growth medium after 8 d (D8) of adipogenic differentiation to induce dedifferentiation and were counted as dedifferentiation day 0 (DD0, equally to D8). The cells are set up into several groups, including with or without insulin, with or without inhibition of the insulin signal. The cells were cultured for 8 d (DD8) to completely induce dedifferentiation. The medium was refreshed every 2–3 d. The insulin signal and insulin-like growth factor 1 signal of the adipocytes can be completely blocked by treatment of the cells with 0.3 µM OSI-906 (linsitinib, from Sellckchem, U.S.A., S109107) [14,15].

### Adipogenic redifferentiation and osteoblast transdifferentiation of the dedifferentiated cells

**Figure 1.**
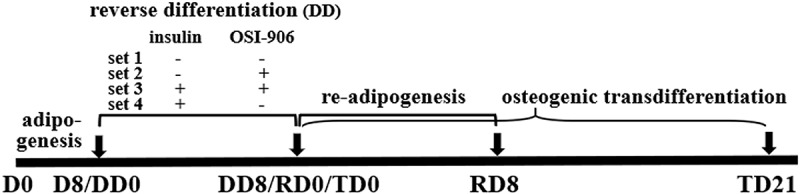
Study design. Mice preadipocytes were first cultured in an adipogenic cocktail for 8 d (D0 to D8/DD0) and then the cells were separated into several sets. Set 1, the adipogenic medium was switched to common cell growth culture medium to continue culturing the adipocytes for 8 d (DD8), leading the cells to slowly dedifferentiation ‘spontaneously’, since lack of exogenous insulin although there is still a tiny amount of endogenous insulin in the medium (i.e. it exists in serum), such that the insulin signalling may be insufficient in the adipocytes; Set 2, used same medium as set 1 but additionally supplemented 0.3 μM of OSI-906 (linsitinib) to completely block insulin signal; Set 3, continues use adipogenic medium but supplemented OSI-906 to completely block insulin signal; Set 4, continues the use of adipogenic medium without OSI-906 for 8 d (DD8). From a time point of DD8 (equally RD0 and TD0), the dedifferentiated cells were then recultured in the common cell growth medium supplemented with insulin alone for adipogenic redifferentiation (RD0 to RD8) or transdifferentiation into osteoblasts by stimulatory supplements for 21 d (TD21). The cells were harvested at D0, D8, DD3, and DD8, respectively, for further analyses

To test whether the dedifferentiated cells can regain stem cell-like ability, the cells were re-induced for adipogenic redifferentiation (RD) using a cell growth medium containing 17 nM insulin that were counted as DD8/RD0 culture for 8 d (RD8). On the other hand, the dedifferentiated cells were induced for osteoblast transdifferentiation (TD) using osteogenic stimuli (cell growth medium supplement with 0.1 μM Dex, 10 mM β-glycerophosphate, and 50 mM ascorbic acid; Sigma) to be counted as DD8/TD0 with continuous culturing occurring for 21 d (TD21, see Figure 1). The mineralized deposition in the transdifferentiated cells was detected by Alizarin Red staining to confirm whether the cells were transdifferentiated into osteoblasts.

### Lipid accumulation and quantification in the cells

Oil red O (ORO) staining method was used to detect and confirm lipid accumulation in adipogenic-differentiated cells and redifferentiated cells. Cells were first fixed in 4% paraformaldehyde and then were stained with ORO solution (60% saturated in isopropanol), photographic using an Olympus 1 × 71 inverted microscope. For the quantification of lipid accumulation, intracellular ORO was extracted using isopropanol, and the absorbance of the obtained supernatant was measured (optical density, OD at 520 nm) and then the content of each sample was determined according to the Lowry method. The data are presented as percentages of appropriate Ctrl (+) at several time points (n ≥ 7) as indicated.

### Quantitative RT-PCR assay

To detect and analyse the mRNA expression profile of target genes, total RNA was isolated from the cells at various time points in each group as previously described [16]. Briefly, 1 μg of total RNA was used for cDNA synthesis using a high-capacity cDNA reverse transcription kit (Toyobo, FSK-100). Quantitative RT-PCR (qPCR) analysis was performed using the Rotor-Gene 3000^TM^ RT-PCR detection system (Corbett Research, Australia) and the SYBR Green I DNA PCR Core Reagent Kit. The amount of transcript was normalized to 18S and averaged from triplicate samples. The primers are shown in Table 1.

### Western blot analysis

The total cellular proteins were isolated from cells at various time points in each group, and immunoblotting was performed as previously described [17]. Briefly, 30 μg of total proteins was separated on SDS-PAGE and then were transferred to a PVDF membrane (GE Healthcare, UK). The membrane was blocked in TBST (20 mM Tris-HCl, pH 7.5, 137 mM NaCl, 0.1% Tween 20) plus 5% de-fat milk and then was incubated with individual primary antibodies against AKT (R & D AF2324), phospho-AKT (Thr308), adiponectin (Abcam), GLUT-4 (Bioworld), SREBP-1 and GAPDH (Santa Cruz Biotechnology) followed by HRP-conjugated secondary antibodies (Jackson). Each band was quantified by densitometry with Image J.

### Statistical analysis

All data were presented as mean ± SEM. Unpaired Student *t* test was used for comparisons between two groups. Two-way ANOVA followed by Tukey’s Multiple Comparison Test was used to compare more than two groups. The differences for the experiments were considered statistically significant at *P* < 0.05. All experiments were repeated at least 7 times.

## Results

### Lack of exogenous insulin leads to the dedifferentiation of adipogenic-differentiated cells

**Figure 2.**
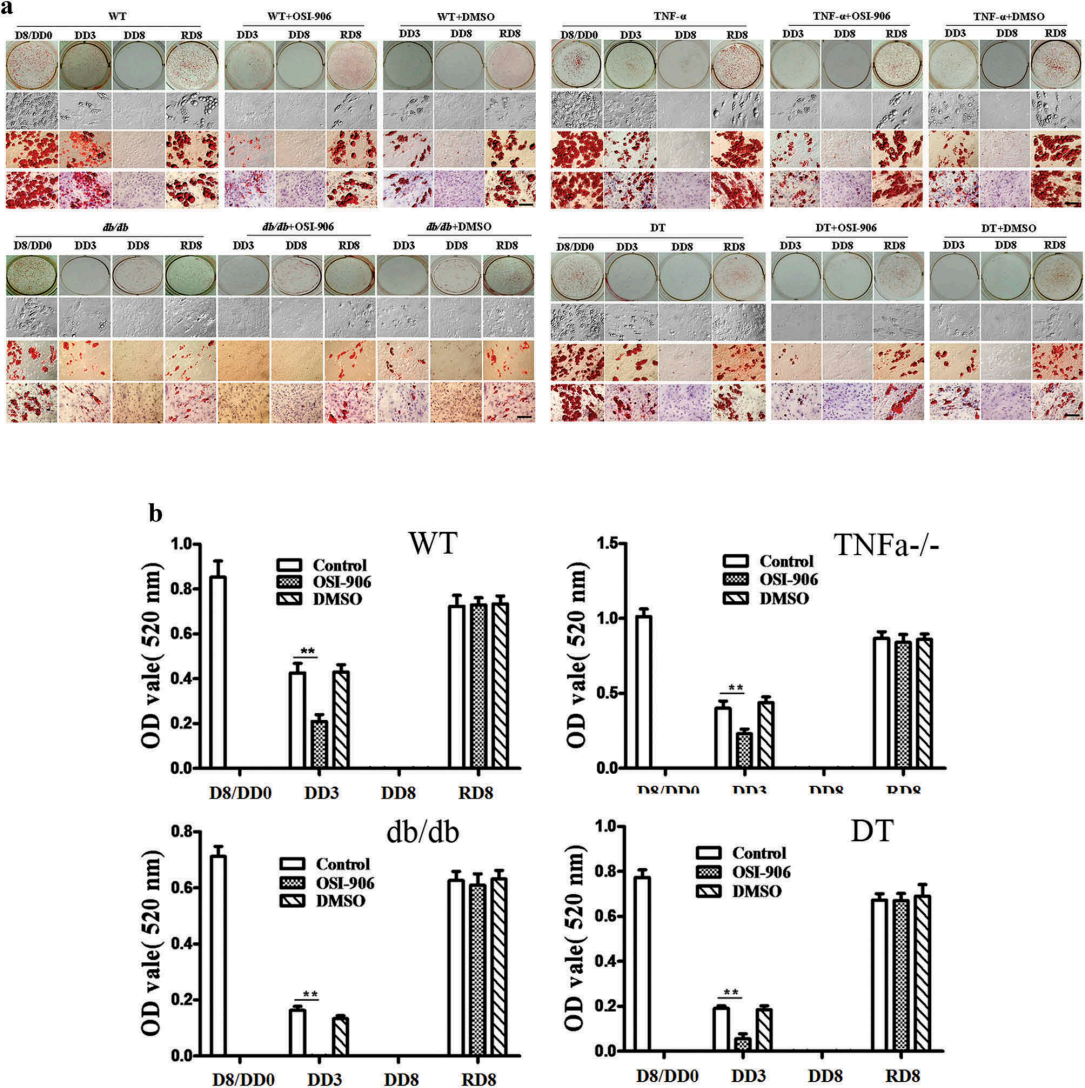
Morphological changes in the WT, TNFα-/-, *db/db* and DT adipocytes at different time points under various conditions as indicated. D8/DD0, the final day of adipogenic differentiation and time point of beginning for dedifferentiation; DD3 and DD8, early and final day of the dedifferentiation of adipogenically differentiated cells, respectively; RD8, the final day of the adipogenic redifferentiation of dedifferentiated cells. As a characterizatic of dedifferentiation, starting from DD1 the adipocytes slowly lose intracellular lipids. The cell morphology was reversed to fibroblast-like shape at DD8 and almost no lipid droplet compared to that of undifferentiated preadipocytes. Scale bar = 20 μm (a). Quantitative analysis of intracellular lipids (b) which equally with the data of ORO staining. DMSO treated cells was used as a control because it was used as a solvent for OSI-906 (0.1% v/v). The results showed no significant effect on the adipogenic differentiation and dedifferentiation of the cells. The data are represented as the means ± SEM, n ≥ 7. **p < 0.01

Figure 1 shows the time and grouping of the experiment. To test the role of insulin on adipocyte dedifferentiation, we first compared the differences in morphology for the four genotype adipocytes under the following conditions: 1) neither without supplement exogenous insulin nor without inhibition of insulin signal; 2) without exogenous insulin but with inhibition of insulin signal; 3) contain exogenous insulin but inhibition of insulin signal at same time; and 4) contain exogenous insulin without inhibition of insulin signal. ORO staining indicated that preadipocytes differentiated to mature adipocytes containing numerous lipid droplets in the cytoplasm (Figure 2(a), D8/DD0). However, the process of adipogenic differentiation was reversed after switching the adipogenic maintenance medium to common cell growth medium, the cells underwent loss of the lipid droplets from DD1 to DD5. The cells were shown to have fibroblast-like shapes, without lipid droplets, and were similar to that of undifferentiated preadipocytes (Figure 2(a), DD8). The above dedifferentiation of the adipocytes can be observed in four genotypes of cells without significant differences of degree among the genotypes (Figure 2(b)).

### Blocking the insulin signal can accelerate the dedifferentiation of the adipocytes

The dedifferentiation begins earlier in the insulin signal-blocked groups compared to that of insulin signal-intact groups, and the cells have completely reversed to the undifferentiated status before DD6 (versus DD8 of intact groups). This is true regardless whether the adipocytes were cultured in an adipogenic maintenance medium (containing exogenous insulin) or if they were cultured in common cell growth medium (without exogenous insulin) (Figure 2(a), see the set indicated with +OSI-906).

### Blocking the insulin signal influences the transcription and translation of adipogenic

#### Differentiation-related molecules

**Figure 3.**
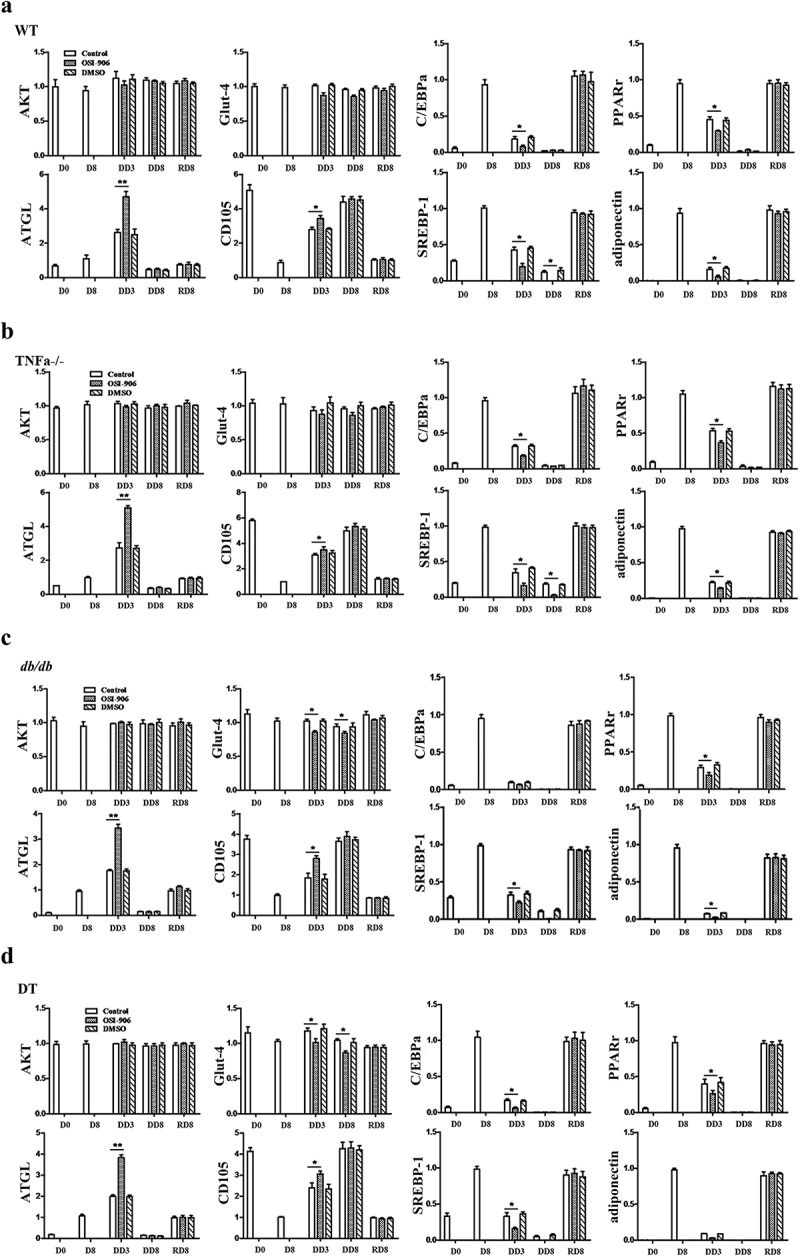
The mRNA expression profile of genes related to insulin receptor signalling, adipogenesis and lipolysis in WT (a), TNFα-/- (b), *db/db* (c) and DT (d) cells. The data are expressed relative to the control (OSI-906 untreated group, and 0.1% DMSO supplemented) cells at each time point. The target gene transcription level was detected by qPCR. The data are presented as the means ± SEM; n ≥ 5 individual experiments. *p < 0.05 and **p < 0.01 relative to the control group

The mRNA expression profile of adipogenic-related target gene corresponding to the time points of cell morphology was investigated by qPCR. The results show that C/EBPα, PPARγ2, adipoQ and SREBP-1 were at basal level at stage D0, but were up-regulated during adipogenic differentiation, while they were significantly down-regulated during dedifferentiation (Figure 3(a–d)). It is worth noting that after removed insulin from a medium and/or there was on inhibition of the insulin signal, the expression level of these genes was significantly down-regulated when compared to insulin signal-intact cells. These adipogenic genes were up-regulated again during the dedifferentiation of adipocytes. Conversely, ATGL, an enzyme that catalyses the rate-limiting hydrolysis step of triglycerides (TG), was up regulated during the dedifferentiation, and reached highest level at time point DD3. It was higher in the insulin signal-inhibited groups when compared to insulin signal-intact groups, but it showed to a lower level at other time points. There was no substantial difference in the mRNA levels of Glut-4 and AKT in the stage of differentiation, dedifferentiation and redifferentiation among the four groups of cells, regardless of whether the insulin signal is intact or inhibited. CD105, one marker for stem cells, was expressed at the highest level at D0, but down-regulated with adipogenic differentiation and up-regulated again during dedifferentiation. This data revealed that the expression patterns of adipogenic, lipolytic and stem cell-related genes at the transcription level coincide with cell morphological dynamic change. Additionally, the dynamic alteration of the expression tendency of the above genes was not significantly different among the four genotypes of cells (Figure 3(a–d)).

**Figure 4.**
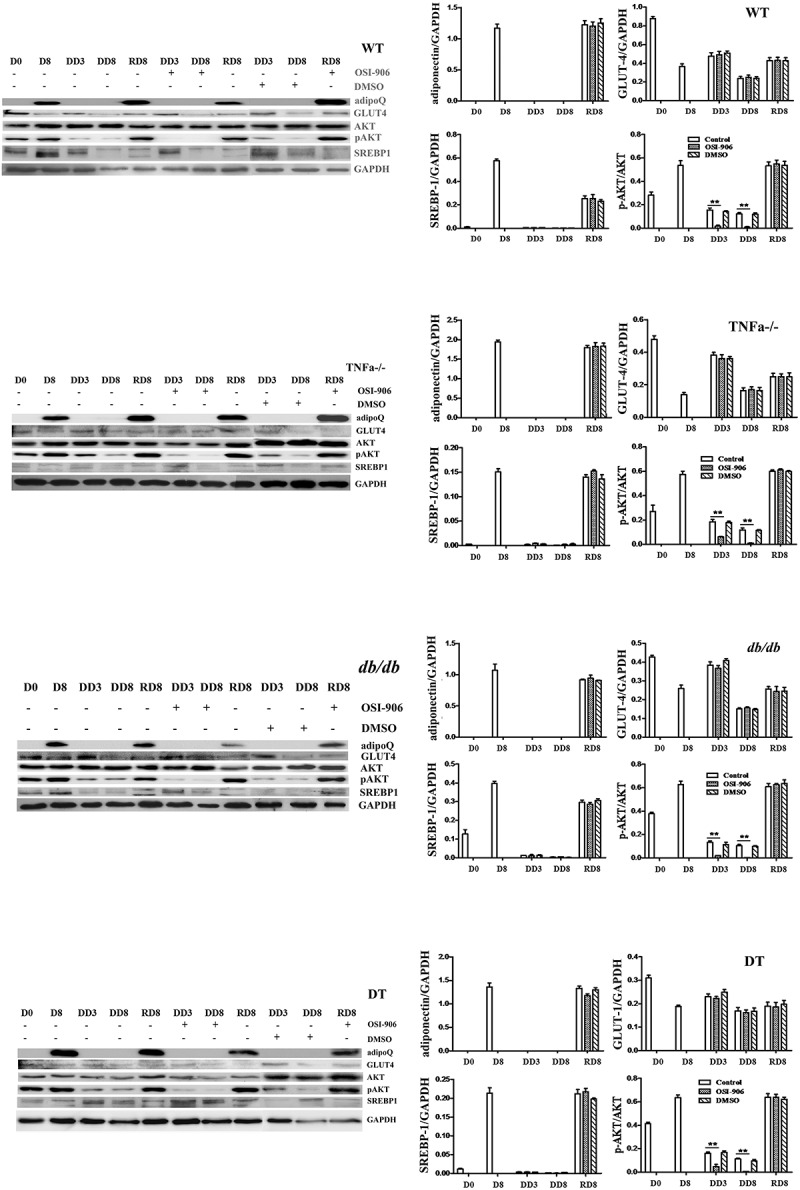
Protein expression levels of adipogenic molecules and insulin receptor signalling markers in WT, TNFα-/-, *db/db* and DT cells. The protein levels of adipoQ, GLUT4, total AKT, SREBP1, and pAKT were analysed by Western blotting. D0, initiation day of adipogenic differentiation; D8, the final day of adipogenic differentiation; DD3 and DD8, early and the final day of the dedifferentiation of adipogenic-differentiated cells, respectively; RD8, the final day of adipogenic redifferentiation. The cells were treated with or without OSI-906 as indicated (left panel). Quantitative analysis (right panel). The data are represented as the means ± SEM; n = 5 individual experiments. **p < 0.01 relative to the control group

To further validate the alteration of related molecules in their translation levels, the protein expression of adipoQ, GLUT-4, SREBP-1, total AKT, and the level of pAKT was detected by Western blotting. As shown in Figure 4, adipoQ expression was at a high level during adipogenic differentiation and redifferentiation, regardless of whether these cells were treated with or without OSI-906 during the previous dedifferentiation. SREBP-1 was at the basal level on the stage of undifferentiation (D0) but was up-regulated during adipogenic differentiation (D8) and redifferentiation (RD8). However, it was down-regulated during dedifferentiation. Total AKT was not significantly changed during all stages. However, the ratio of pAKT/AKT increased upon adipogenic differentiation and redifferentiation, while it significantly decreased during dedifferentiation (Figure 4, RD8). Its ratio was the lowest (closely to zero) in insulin signal-inhibited groups, indicating that insulin signalling is nearly completely blocked. GLUT-4 showed no significant differences among the four genotypes of cells at the same stage, although it was shown to have certain dynamic changes during the processes of differentiation, dedifferentiation and redifferentiation. The above observations in all four genotypes of cells were without significant differences.

### Dedifferentiated adipocytes regained adipogenic redifferentiation and

#### Transdifferentiation ability

**Figure 5.**
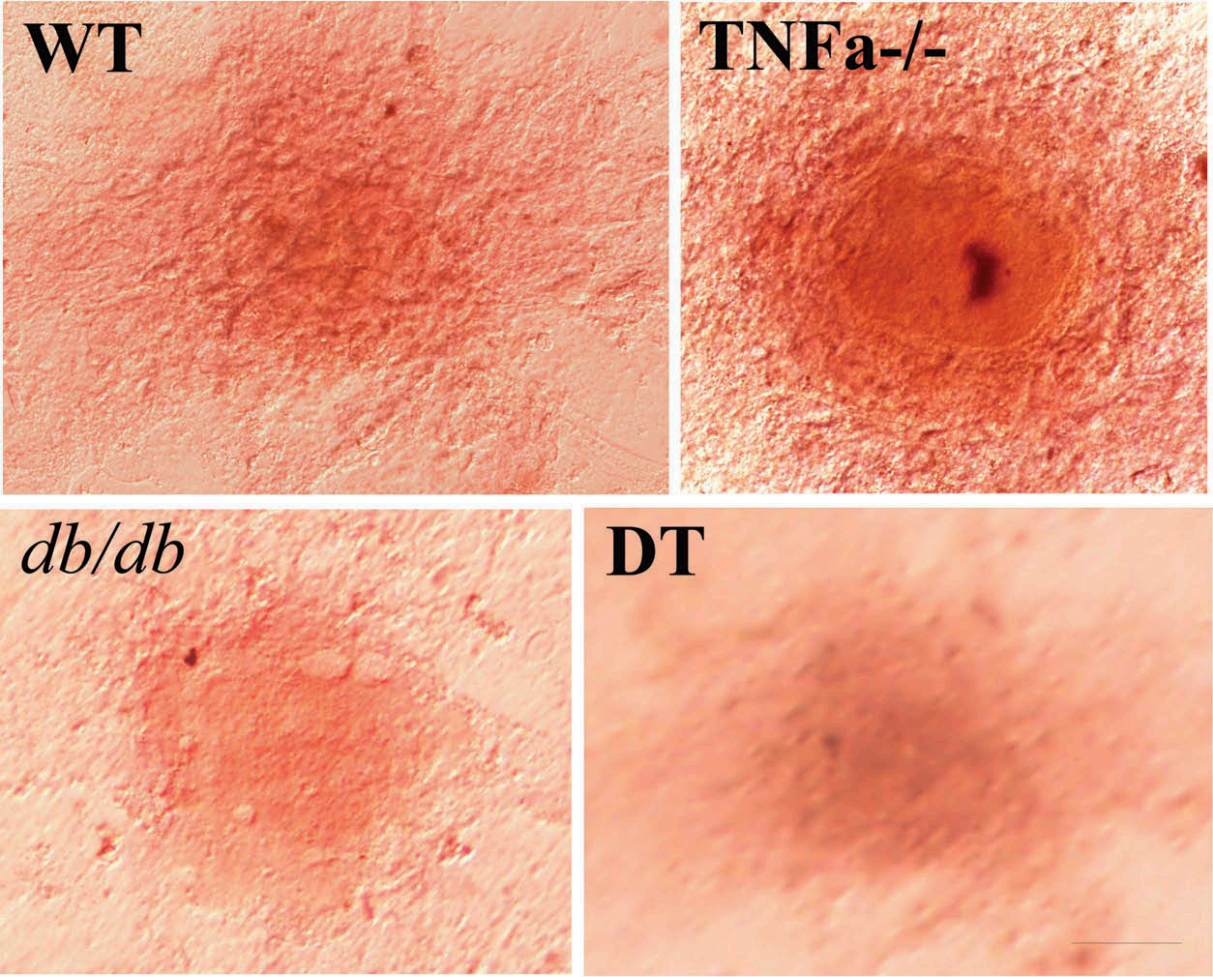
Mineralized deposition in the WT, TNFα-/-, *db/db* and DT transdifferentiated cells was detected by Alizarin Red staining. The result showed positive staining of cell structure, indicating the dedifferentiated cells can transdifferentiation to osteoblast. The bar indicates 50 μm

Four genotypes of cells of the dedifferentiated not only have the ability to redifferentiate into adipocytes (Figure 2–4) but also have the ability to transdifferentiate to other cell type. Alizarin red staining indicates that the dedifferentiated cells can transdifferentiate into osteoblasts under suitable inducing conditions (Figure 5). No significant difference was found in the above dynamic change tendency and degree of change among the four genotypes of cells.

## Discussion

The phenomena of cell differentiation, dedifferentiation and reprogramming are the basis of tissue repair and regeneration [18–20]. However, the triggers and regulatory mechanisms of adipocyte dedifferentiation remain unclear, even though the phenomenon of adipocyte dedifferentiation has been reported earlier [1,21–24]. This study demonstrates the importance of insulin/insulin signal in adipocyte dedifferentiation. Our data indicate that the lack of insulin in the cell culture medium is responsible for the phenotype switch in mouse adipocytes. We observed that adipogenic differentiation was paused initially due to the lack of continuous insulin induction. The process of adipogenesis can shift to dedifferentiation, if without insulin treats the cells again and continually. Moreover, the progression of the dedifferentiation was significantly promoted after the inhibition of insulin/IGF-1 signal by OSI-906, since it effectively blocked both the AKT and MAP kinase pathways in adipocytes [14,15].

It is known that humans and other animals adipose tissue-derived MSCs can differentiate into adipocytes spontaneously in vitro during culture in a common cell growth medium, although at a lower ratio. Such type of spontaneous adipogenic differentiation of stromal cells in vitro may be its responses to the stimulation of the insulin that exists naturally in serum. As a nutrient that contains growth factors, serum usually is added to the cell culture medium to promote cell proliferation. In the present study, we confirmed that the presence or absence of insulin in the medium is a key causal issue in determining whether differentiated cells maintain a mature phenotype or turnover towards dedifferentiation. Therefore, dedifferentiation occurred significantly earlier in the insulin/IGF-1 signal-blocked groups compared to the cells with insulin signal being unblocked. This is due to the fact that a tiny amount of insulin is naturally present in the cell growth medium (i.e. come from 10% of bovine serum), that leads the adipocytes of insulin signal intact (untreated with OSI-906) groups own somehow resistance to dedifferentiation even after removed higher concentration of the exogenous insulin from adipogenic medium.

A recent report showed that visceral preadipocytes isolated from type 2 diabetes patients were more abundant. The patients have more numerous dedifferentiated cells than do control subjects, with higher expression of stem cell markers [25]. Thus, more dedifferentiated or immature adipocytes likely exist in white adipose tissues in individuals with insulin signalling dysfunction such as those with insulin resistance, although detailed work has to be done to confirm this issue. Moreover, whether these dedifferentiated cells can differentiate into more adipocytes in vivo even with slightly dysfunction of insulin signalling or their resulting ultimate fate needs to be further investigated in the future.

Insulin signalling in preadipocytes and adipocytes depends on key signal molecules such as PI-3K and AKT [14,26,27]. Several studies showed that the factors of insulin/IGF-1 signal, such as PIK3R1, AKT3 and MAPK3, were down-regulated during the adipogenic differentiation of hMACs but were up-regulated after removing the adipogenic stimuli from the adipogenic differentiation medium [28,29]. During the dedifferentiation of adipocytes in parallel with the regaining of original biological phenotypes as stem cell-like properties, mature adipocyte gene expression was significantly decreased, but genes related to cell proliferation and dedifferentiation increased [7,19,21]. In the present study, we found that the pAKT level was significantly reduced during dedifferentiation compared with that of the differentiation stage and redifferentiation stage and was more decreased after inhibition of the insulin signal, although total AKT was not significantly altered in transcription and translation levels. This indicates that intact insulin/AKT signalling plays an important role in negatively controlling the dedifferentiation of adipogenic-differentiated cells.

ATGL is expressed predominantly in adipose tissue [30]. In the present study, as expected, ATGL was up-regulated upon dedifferentiation, reached a peak at time point of DD3 in all groups, but it was decreased at the late phase of dedifferentiation (DD8). This result indicates that increased lipolysis is related to the hypofunction of insulin signalling, resulting in up-regulated lipolytic factors such as ATGL, and down-regulated adipogenic factors, eventually promoting the metabolism of intracellular lipids and adipocyte dedifferentiation [30,31].

It is known that adipose tissue in obesity secretes a high level of pro-inflammatory adipokines, such as leptin, TNFα and ILs, but few anti-inflammatory cytokines such as adipoQ. This results in metabolic disorder and chronic inflammation [23,32–34]. TNFα suppresses the expression of genes that are involved in lipid and glucose metabolism such as GLUT4, hormone-sensitive lipase, adipocyte complement-related protein and those encoding transcription factors such as C/EBPα and PPARγ2 [17,35,36]. TNFα-treated adipocytes stimulate lipolysis and the loss of intracellular TG [29,32]. To verify whether mutation of the TNFα gene and leptin receptor (*db*) gene can influence adipocyte dedifferentiation, in vitro adipogenic differentiated cells of *db/db*, TNFα-/- and DT mice were induced for dedifferentiation in this study. The data showed no significant difference in dedifferentiation among these cells, although the cells isolated from TNFα-/- mice showed a higher adipogenic ratio and more easily underwent dedifferentiation than that of WT mice and *db/db* obese mice. We previously found that DT mice exhibited a more obese phenotype than that of age- and gender-matched *db/db* mice when fed with a standard diet (unpublished data). Therefore, our in vitro data can explain the in vivo results above mentioned, at least in part, i.e. TNFα combined with *db* gene mutation can indeed influence adipogenic differentiation and adipocyte dedifferentiation.

Insulin is well known to play a critical role in triggering and maintaining preadipocyte differentiation. Insulin binds to its receptor and then triggers the phosphorylation of IRS, which, in turn, recruits and sequentially activates PI-3K and AKT [14,15], which plays important roles in adipose biology [37,38]. Although insulin is known to stimulate adipocyte differentiation dose-dependently at the early stage of differentiation [39], Konneker et al. recently reported that high concentration of insulin (>3.4 μM) inhibits lipid storage in the late phase of human primary preadipocytes differentiation [40]. Similar result was also can be found in a previous report that long-term exposure to insulin eliciting a more immature phenotype in 3T3-L1 adipocytes [41]. These data indicated a more immature stage of conversion in a high concentration of insulin or after they were treated with insulin for long term. As we know that the biological role of insulin is through its receptors (insulin receptor and IGF-1 receptor) on effector cell surface. However, the status of insulin signalling was not monitored in their works. Therefore, we cannot rule out the existence of insulin resistance in these adipocytes, similar to the changes in adipose tissues in type 2 diabetes patients [25] as we described earlier. In turn, these findings also support our present data from the side, that is, the intact insulin signal negatively regulates adipocyte dedifferentiation. In the present study, we used a low-medium concentration of insulin (17 nM) for induce primary mouse preadipocytes differentiation, as illustrated in the most papers. We also found that primary human adipocytes shown more resistance to insulin signal dysfunction than those cells from mice (unpublished data).

Finally, our results indicate that exquisite control of the concentration of insulin (control nutrients intake) or function of insulin signalling (certain natural compounds) may be a new potential strategy to regulate preadipocyte differentiation, adipocyte dedifferentiation and obesity development. On the other hand, our findings may also contribute to enriching our understanding of the roles of insulin in adipose tissue development and remodelling. Specific changes in the environmental or hormonal milieu may regulate adipocytes or other types of cells to withdraw from the cell cycle and then undergo dedifferentiation and acquire stem cell characteristics. We have found supporting evidence of this from recent reports [42–44]. These dedifferentiated cells have also been shown to immunomodulate the microenvironment and secrete abundant growth factors which minimizes inflammation and contribute to repair and regeneration [45].

## Conclusions

This data indicates that: 1) adipogenic differentiated mature adipocytes can reverse to the undifferentiated status if it without continuous induction by insulin or under a situation of insulin resistance; these dedifferentiated cells can regain multilineage differentiation potentials similar to MSCs; 2) intact insulin signal is essential to maintaining the phenotype of mature adipocytes, and it plays a key role in inhibiting dedifferentiation of the adipocytes, may acts as a key player in the negative feedback loop control this process; 3) the event of dedifferentiation of the adipocytes was independent of the cell genotypes, but is one widespread biologic phenomenon in adipocytes. Our current data demonstrate that in addition to its role in inducing adipogenic differentiation and mature phenotype maintenance, the intact insulin signal is a crucial regulatory pathway that orchestrates the balance of adipogenic differentiation and dedifferentiation via PI-3K/AKT signal pathway. Therefore, proper control of insulin level and insulin signalling could be beneficial to maintain the balance between the differentiation and dedifferentiation of adipocytes to prevent obesity and its related disorders. Further study may be needed to identify the roles of other pathways and their molecular mechanisms on adipocyte dedifferentiation, such as mTOR signal [46]. Moreover, the study of lipid metabolome [47] of adipocytes in different stages of dedifferentiation will further provide the characteristics of metabolism and regulation of molecules in the process of adipocyte dedifferentiation that help to better understand mechanism on adipocyte reprogramming.
